# Identifying cellular senescence associated genes involved in the progression of end-stage renal disease as new biomarkers

**DOI:** 10.1186/s12882-023-03285-0

**Published:** 2023-08-08

**Authors:** Yu-jia Xi, Qiang Guo, Ran Zhang, Guo-sheng Duan, Sheng-xiao Zhang

**Affiliations:** 1 Department of Urology, Second Hospital of Shanxi Medical University, Taiyuan, Shanxi Province China; 2 Key Laboratory of Cellular Physiology at Shanxi Medical University, Ministry of Education, Taiyuan, Shanxi Province China; 3 School of Public Health, Shanxi Medical University, Taiyuan, Shanxi Province China; 4 Fifth School of Clinical Medicine, Shanxi Provincial People’s Hospital, Shanxi Medical University, Taiyuan, Shanxi Province China; 5 Department of Rheumatology, Second Hospital of Shanxi Medical University, 382 Wuyi Road, Taiyuan, 030001 Shanxi Province China

**Keywords:** ESRD, Cellular senescence, Immune regulation, GEO, Bioinformatics

## Abstract

**Background:**

Cellular senescence plays an essential role in the development and progression of end-stage renal disease (ESRD). However, the detailed mechanisms phenomenon remains unclear.

**Methods:**

The mRNA expression profiling dataset GSE37171 was taken from the Gene Expression Omnibus (GEO) database. The cell senescence-associated hub genes were selected by applying protein–protein interaction (PPI), followed by correlation analysis, gene interaction analysis, Gene Ontology (GO), and Kyoto Encyclopedia of Genes and Genomes (KEGG) pathway enrichment analysis. We next explored the relationships of hub genes with miRNAs, TFs, and diseases. The absolute abundance of eight immune cells and two stromal cells were calculated by MCPcount and the correlation of hub genes with these ten cells was analyzed. Lasso was used to selecting for trait genes. ROC curves and DCA decision curves were used to assess the accuracy and predictive power of the trait genes.

**Results:**

A total of 65 cellular senescence signature genes were identified among patients and controls. The PPI network screened out ten hub genes. GO and KEGG indicated that ten hub genes were associated with ESRD progression. Transcription factor gene interactions and common regulatory networks of miRNAs were also identified in the datasets. The hub genes were significantly correlated with immune cells and stromal cells. Then the lasso model was constructed to screen out the five most relevant signature genes (FOS, FOXO3, SIRT1, TP53, SMARCA4). The area under the ROC curve (AUC) showed that these five characteristic genes have good resolving power for the diagnostic model.

**Conclusions:**

Our findings suggested that cellular senescence-associated genes played an important role in the development of ESRD and immune regulation.

**Supplementary Information:**

The online version contains supplementary material available at 10.1186/s12882-023-03285-0.

## Background

End-stage renal disease (ESRD) is commonly referred to as uremia. It is a clinical syndrome consisting of a series of symptoms and metabolic disturbances caused by various renal diseases, leading to a progressive and irreversible decline in renal function until loss of function and eventual development of ESRD [1]. The glomerulus undertakes the filtration of blood in the circulatory system, and structural destruction or decreased glomerular filtration rate is the direct cause of these pathophysiological changes. ESRD has a prevalence of up to 1 in 100,000 and can have a variety of clinical complications. Aging is associated with significant changes in the structure and function of the kidney, even in the absence of age-related complications [2]. The various functions of the kidney are affected by the complex process of aging, which manifests itself in the decline and loss of kidney structure and function [3]. Aging is accompanied by an increased incidence of various cardiovascular diseases, such as atherosclerosis and hypertension, which indirectly lead to renal failure [4]. Renal failure, whether caused by direct or indirect factors, may eventually progress to end-stage renal failure, and it is, therefore, important to explore the mechanisms of aging-induced renal failure to develop new therapeutic strategies to reduce the burden of renal function due to failure. Aging-based therapies are currently being sought to treat and prevent ESRD in humans, however, insight into the mechanisms of cellular senescence genes in the development of renal failure and elucidation of the interconnections between aging and ESRD are needed, which will enable us to develop more effective therapeutic strategies to reduce the burden of ESRD.

**Fig. 1:**
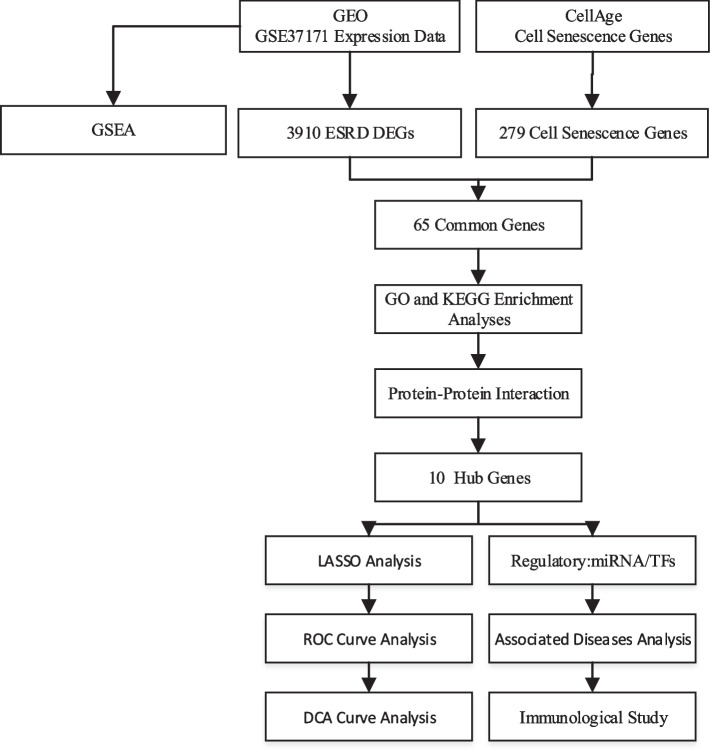
Schematic illustration of the overall general workflow of this study

In the era of genomics, gene chips have been widely used to explore the mechanisms of disease, bringing some new insights into pathogenesis at the genetic level. However, to date, few studies have explored the interaction of aging-related genes with immune regulation in ESRD. Therefore, this study aims to identify the link between ESRD and cellular senescence from the perspective of bioinformatics analysis and to provide a reference for further research on ESRD. The research process and methodology of this paper are illustrated in the form of a flow chart in Fig. 1.

## Methods

### Data acquisition

Gene expression profiles were obtained from the Gene Expression Omnibus (GEO) database (http://www.ncbi.nlm.nih.gov/geo). This database is a database repository of high-throughput gene expression data, hybridization arrays, chips, and microarrays [5]. The GSE37171 microarray dataset in the GEO database was selected for follow-up analysis, where 75 patients with ESRD were studied as an experimental group against a healthy sample of 40. A 3:1 case–control design was used to compare gene expression between patients with end-stage renal failure and healthy controls (HCs). Patients aged 18 to 75 years with stage five renal disease waiting for renal transplantation had stable clinical characteristics and were provided written informed consent to be included in the study. The ethical approval and informed consent statements for this data have been clarified in the original text [1], and we do not need to make any additional statements. All samples were taken from whole blood in the early morning, fasting, clinically stable, and without receiving immunosuppressive drugs. Patients were treated according to the Canadian Chronic Nephropathy Guidelines. The age and sex of HCs were comparable to those of the screened patients to ensure that there were no known diseases or drug treatments.

CellAge (http://genomics.senescence.info/cells) is a manually curated database that contains 279 human genes that drive cellular senescence and performs various comprehensive analyses. A total of 279 genes related to cellular senescence were selected through the CellAge database.

### Differential expression analysis

Differentially expressed genes (DEGs) were screened using the “limma” package in the R package. DEGs for ESRD were obtained by testing and screening the dataset through a critical threshold (adjust *P* < 0.05 and | logFC |> 0.585). Data were analyzed by sangerbox 3.0 (http://vip.sangerbox.com/home.html), the online website that analyzes the overlap between differential genes and cellular senescence characteristic genes in ESRD while visualizing the results. The intersection genes were the potential genes studied for further study analysis.

### Enrichment analysis of DEGs associated with cellular senescence

Gene set enrichment analysis is an important analytical effort to classify common biological insights, such as biological processes or chromosomal locations associated with different interrelated diseases. GSEA (Gene Set Enrichment Analysis): Gene set enrichment analysis is an enrichment method, which contains a variety of annotation files such as GO, KEGG, Reactome, etc., and also provides the corresponding analysis software and a gene set database [6]. Gene Ontology (GO) is a widely used ontology in the field of bioinformatics [7, 8], which covers three aspects of biology: cellular components, molecular functions, and biological processes. KEGG (Kyoto Encyclopedia of Genes and Genomes) is a database for genome deciphering [9]. It is one of the most commonly used bioinformatics databases internationally and is known as the "repository of advanced functions and utilities for understanding biological systems". GO, KEGG, and GSEA enrichment analysis was performed using the R package clusterProfiler, and the GO and KEGGenrichment results were visualized as a pathway network map using the R package “enrichplot”. GSEA enrichment analysis results are visualized as a mountain range map by the “enrichplot” package.

### Protein–protein interaction analysis

Protein–protein interaction (PPI) networks are based on the Search Tools for Retrieving Interacting Genes (STRING) database (https://string-db.org). It was derived that this database covers almost all functional interactions between expressed proteins, and interactions with a composite score > 0.4 were considered medium confidence [10]. The results of this analysis were visualized using Cytoscape (version 3.9.1). The topology of the network was analyzed by the “CytoHubba” plugin, and the top 10 nodes in the Degree ranking of the network were selected as Hub genes for subsequent analysis. Subsequently, hub genes were analyzed in-depth in multiple directions: GO functional annotation, KEGG pathway enrichment, and visualization were done using "clusterProfiler" R packages; their feature correlation coefficients were calculated at the R "cor" function and the correlation heatmap was drawn with the help of the "corrplot" R package by Pearson correlation test; and in GENEMANIA (http://genemania.org/search/), was used to construct a protein–protein interaction network for hub genes to assess the function of hub genes.

### Network relationship between hub genes and miRNA, TFs

Data were analyzed using Networkanalyst (https://www.networkanalyst.ca/). The online platform was used to search for hub genes-related miRNAs in the miRTarBase and TarBase databases, respectively, and hub gene-related transcription factors (TFs) in the JASPAR database. The relationship between hub genes and miRNAs, and TFs were all visualized through Cytoscape.

### Hub gene-related disease analysis

Potential complications associated with end-stage renal disease and cellular senescence were investigated according to hub genes in the DisGeNET database. DisGeNET integrates the existing database with the disease gene association information obtained from the literature through machine learning and builds a new database containing the disease, mutation, and gene association information [11, 12].

### Analysis of immune cell and immunity regulation

MCPcounter is a validated computational method that can robustly quantify the abundance of multiple immune and non-immune matrix populations of heterogeneous tissues (such as normal or malignant tissues), that is, MCPcounter targets tumor or non-tumor tissues, and the species is human. This method allows robust quantification of the absolute abundance of eight immune cell populations and two stromal cell populations in heterogeneous tissues from transcriptomics data [13]. Immune abundance and matrix abundance were estimated using the R package “MCPcounter”, in combination with expression profiles.

### Screening of trait genes and assessment of predictive power

To further investigate the characteristic genes, LASSO penalized COX regression analysis was performed using the R “glmnet” package, and classifiers were constructed and validated by a binomial method. Based on clinical survival outcomes and prediction results, ROC curves were plotted using the R “pROC” package to assess the individual prediction accuracy of each gene.

### Statistical analysis

Statistical analyses were performed using R software (version 4.1.2). The Pearson correlation test was used to analyze the correlation of the 10 hub genes. The area under the ROC curve AUC value was used to assess the prediction accuracy of the hub gene. Use of non-parametric tests to calculate differences in immune cell content between diseased and normal controls, and *p* < 0.05 was considered significant.

## Results

### Screening of DEGs associated with cellular senescence and GSEA enrichment analysis

**Fig. 2:**
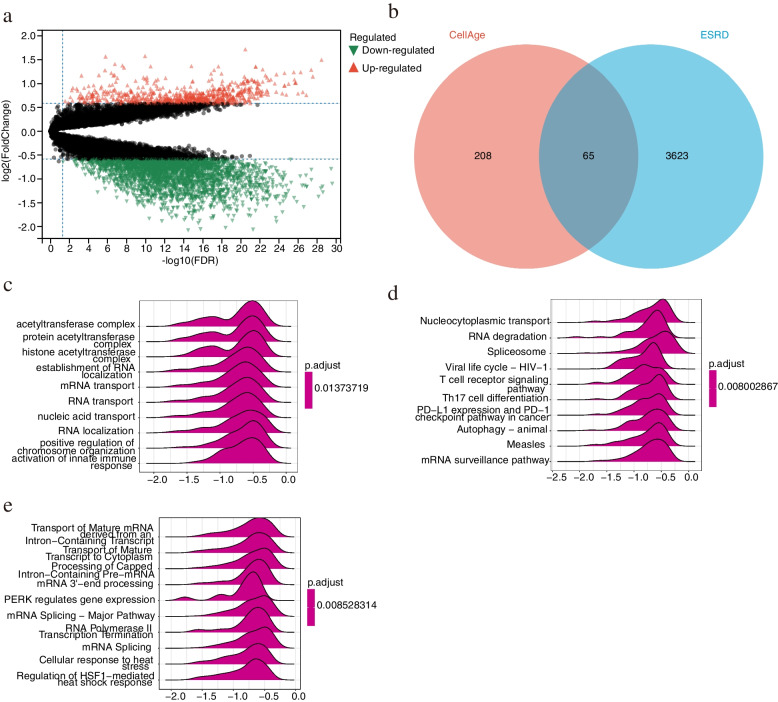
**a** Volcano plot of up-and down-regulated differential genes in ESRD (adjust *P* value < 0.05), with red points indicating up-regulated DEGs; green points indicating down-regulated DEGs. **b** Venn diagram. List1: Differentially expressed genes selected from GSE37171. List2: Cellular senescence-associated genes downloaded from CellAge database. **c**-**e** GSEA enrichment analysis

After pre-processing the cohort in the GSE37171 dataset, 3910 DEGs (788 up-regulated and 3122 down-regulated genes) for advanced kidney disease were screened by a critical threshold (adj. *p*-value < 0.05 and | logFC |> 0.585). Visualization of the differential analysis of GSE37171 is shown in Fig. 2a. Venn diagram showed that 65 genes were hybridized between DEGs in ESRD and cellular senescence-related genes (Fig. 2b). The results of GSEA analysis of the Go database showed that most of the genes were mainly associated with acetyltransferase complex, histone acetyltransferase complex, the establishment of RNA localization, and mRNA transport (Fig. 2c); GSEA enrichment analysis of the KEGG database was mainly associated with nucleocytoplasmic transport, T cell receptor signaling pathway, Th17 cell differentiation, PD-L1 expression and PD-1 checkpoint pathway in cancer (Fig. 2d). GSEA analysis of the Reactome database was mainly associated with the transport of Mature mRNA derived from an Intron − Containing Transcript, transport of Mature Transcript to Cytoplasm, processing of Capped Intron − Containing Pre − mRNA (Fig. 2e).

### GO enrichment analysis and KEGG pathway enrichment analysis of differential expression genes associated with cellular senescence

**Fig. 3:**
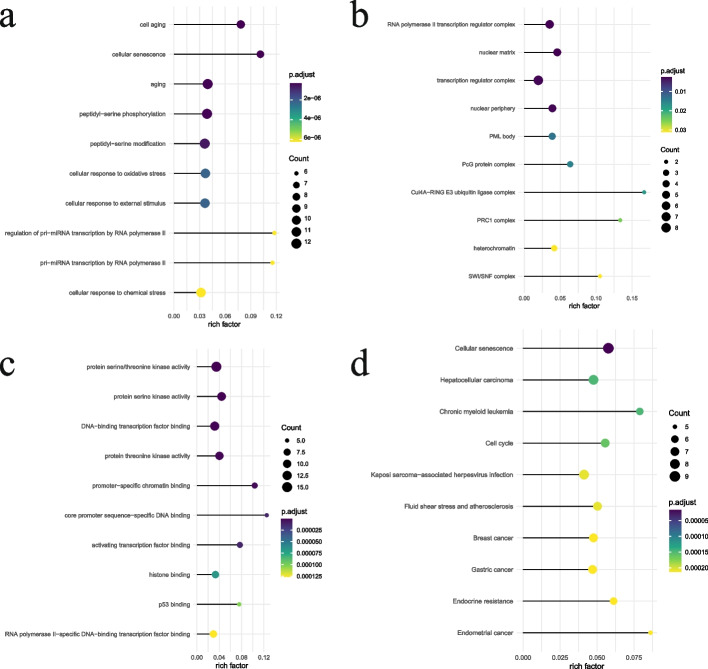
**a**-**c** The results of GO functional enrichment (BP, CC, and MF) were done for the common association genes between ESRD and cellular senescence, and their results were all expressed as p. adjust sort; color indicates adj *P* value, circle size indicates the number, the abscissa is a rich factor. **d** Bubble plots of the results of enrichment of KEGG signaling pathways were done for common association genes between ESRD and cellular senescence. Circle size indicates count number, color indicates negative logarithm with adj *P* value base 10, and the abscissa is gene ratio

The results of GO functional enrichment (BP, CC, and MF) showed that BP was mainly enriched for the regulation of cell aging, cellular senescence, aging, cellular response to oxidative stress, and so on (Fig. 3a). CC was mainly associated with RNA polymerase II transcription regulator complex, nuclear matrix, nuclear periphery, PML body (Fig. 3b). and MF was linked to enzymes such as protein serine/threonine kinase activity, protein serine kinase activity, DNA − binding transcription factor binding (Fig. 3c). KEGG functional enrichment mainly showed biological pathways related to cellular senescence, cell cycle, and endocrine resistance, in addition to hepatocellular carcinoma, breast cancer, gastric cancer, Kaposi sarcoma-associated herpesvirus infection, fluid shear, stress, and atherosclerosis were also included in the collected results, which seems to suggest a potential link between these diseases and end-stage renal failure (Fig. 3d).

### Construction of protein–protein interaction network and screening and analysis of hub genes and enrichment analysis

**Fig. 4:**
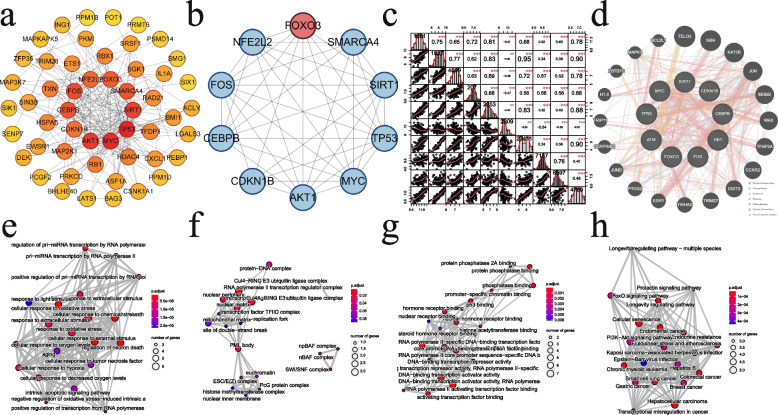
**a** Protein–protein interaction network of 65 genes imported from the String database and hiding disconnection points. **b** The genes with the top ten Degree values were selected as Hubs according to CytoHubba's built-in algorithm. **c** The correlation heatmap of the ten hub genes. The numbers in the squares running across the diagonal represent the EntrezID of the gene in the heat map. **d** Gene–gene interaction network of ten hub genes, showing 20 most frequently changing neighboring genes. Each node represents a gene. The color of the edges reflects the roles of physical interactions, co-expression, prediction, pathway, co-localization, genetic interactions, and shared protein domains, respectively. **e**–**h** Enrichment results of 10 Hub genes in GO functional annotation (BP, CC, and MF) and KEGG signaling pathway enrichment results

A total of 65 ESRD-associated senescence signature genes were introduced into were imported into the Cytoscape data platform for visualization operation, and the resulting PPI relationship network had 52 nodes with 224 edges (Fig. 4a). Under Cytoscape platform network topology analysis, the ten genes calculated according to the Degree were TP53, MYC, AKT1, SIRT1, FOS, CEBPB, SMARCA4, FOXO3, NFE2L2, CDKN1B (Fig. 4b). Correlation analysis was done for the ten hub genes, and the results showed that there was a significant positive correlation between the nine genes down-regulated in the expression profile of ESRD, and a significant negative correlation with the up-regulated gene FOXO3 (Fig. 4c). Among the various types of interactions shown by the gene–gene interaction network, the top two were in physical interactions and co-expression, accounting for 61.93% and 14.11%, respectively, and the related gene functions were mainly related to the positive regulation of transcription by RNA polymerase II, intrinsic apoptotic signaling pathway, response to oxidative stress, and cellular response to chemical stress (Fig. 4d).

The results of GO functional annotation and KEGG pathway enrichment analysis are reflected in Fig. 4E and F. The enrichment results of ten hub genes in GO functional annotations (BP, CC, and MF) showed that BP was associated with oxidation, such as oxidative stress, reactive oxygen species response, cellular response to hypoxia, cellular response to reduced oxygen levels, negative regulation of oxidative stress-induced intrinsic apoptotic signaling pathways, response to oxidative stress, cellular response to oxidative stress (Fig. 4e); CC was mainly enriched in protein − DNA complex, RNA polymerase II transcription regulator complex (Fig. 4f). and MF was associated with DNA − binding transcription activator activity, hormone receptor binding (Fig. 4g). The results of the KEGG pathway enrichment analysis showed that ten hub genes were mainly associated with the FoxO signaling pathway, Endocrine resistance, PI3K − Akt signaling pathway, and related cancer pathways (Fig. 4h).

### Construction of network relationship between hub genes and microRNA, transcription factor, and diseases

**Fig. 5:**
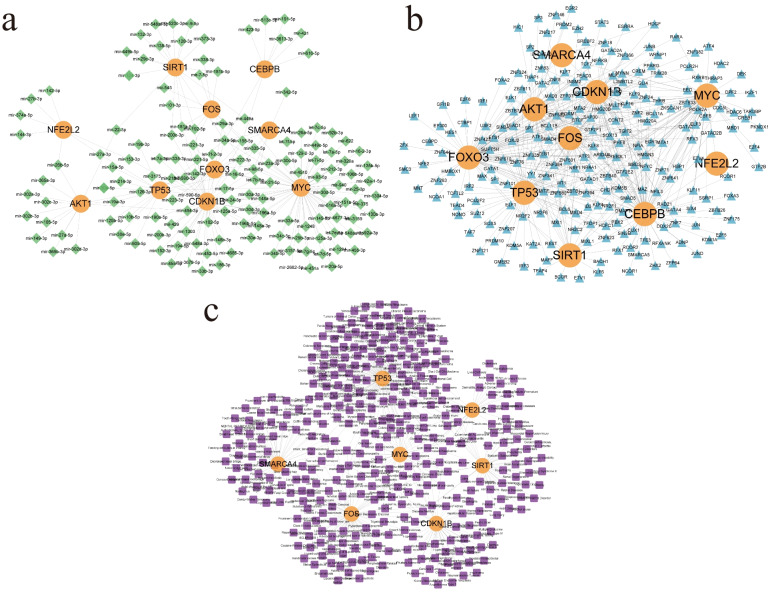
**a** miRNA-gene regulatory network constructed by miRTarBase and TarBase database screening. **b** TF-gene regulatory network was constructed by screening in ENCODE database. **c** Related diseases that may be complicated with ESRD and cellular senescence

Screening hub genes related miRNAs in miRTarBase and TarBase databases, the gene regulatory networks constructed by ten hub genes and their regulated 146 miRNAs are shown in Fig. 5a. The constructed TF-gene regulatory network was screened in the ENCODE database and constituted 231 nodes representing TFs and Hub and 480 edges reflecting their interrelationships (Fig. 5b). In the constructed hub-miRNA regulatory network, hsa-mir-34a-5p and hsa-mir-155-5p belong to the central regulatory miRNAs and are associated with six hub genes for regulatory intervention in kidney diseases. Total 86 TFs can bind to CEBPB core genes and play regulatory roles, and Degree ranks first in the network topology analysis of the hub-TFs relationship network. A total of 359 diseases associated with hub genes were searched in the DisGeNET database, of which CEBPB, AKT1, and FOXO3 gene association network elements constituted less than 3 and separated from other relationships and were not included in the analysis for the time being. In the gene-disease relationship network (Fig. 5c), there are 136 diseases associated with the TP53 gene, mainly covering systemic organs or histiocyte tumors such as liver, lung, and kidney, and TP53 may be a key regulatory gene in the prevention of related terminal renal failure diseases.

### Analysis of immune regulation and immune cell content

**Fig. 6:**
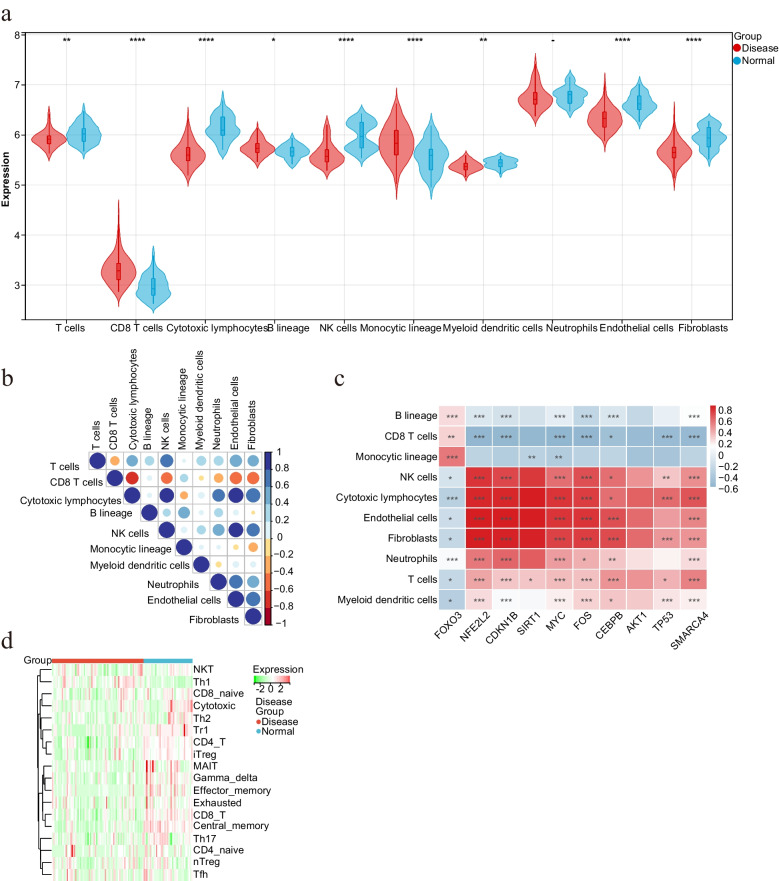
**a** Levels of eight immune cells and two stromal cells in the healthy control and ESRD disease groups. **b** Correlation analysis between eight immune cells and two stromal cells. **c** Heat map of the correlation between the hub genes and eight immune cells and two stromal cells. **d** Absolute abundance of eight immune cells and two stromal cells in ESRD patients and healthy controls

To assess in-depth the differences in immune regulation between ESRD and healthy controls, we calculated the absolute abundance of eight immune cells and two stromal cells using the MCPcount algorithm. The profile and regulation abundance of the eight immune cells and two stromal cells between the control and disease groups were presented as violin plots (Fig. 6a and Table S1). The results showed that compared to healthy controls, ESRD had significantly lower levels of T cells, cytotoxic lymphocytes, NK cells, myeloid dendritic cells, endothelial cells, and fibroblasts, while CD8 T cells, B lineage, and monocytic lineage levels were significantly increased. We then analyzed the correlations between the 10 cells mentioned above and the results are shown in Fig. 6b. The correlations of the ten central genes with eight immune cells and two stromal cells (Fig. 6c) indicated that the central genes were associated with most immune cell infiltrates and two stromal cells, with the down-regulated genes correlating positively with T cells, NK cells, cytotoxic lymphocytes neutrophils, myeloid dendritic cells, endothelial cells, and fibroblasts were positively correlated with B lineage, CD8 T cells, and monocytic lineage. The complete opposite was true for the upregulated gene FOXO3. ImmuCellAI analyzed 18 common T-cell species. The heat map results (Fig. 6d) show that the vast majority of T-cell levels are lower in ESRD patients than in normal controls.

### Screening of trait genes and assessment of predictive power

**Fig. 7:**
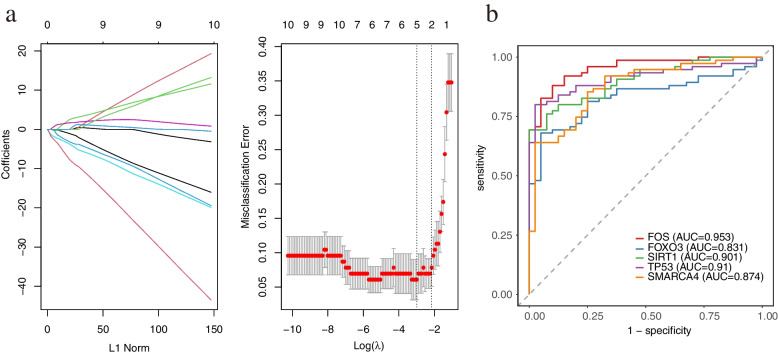
**a** The five feature factors were screened by binomial analysis to construct a classifier under "class" cross-validation. **b** ROC curves show the effectiveness of the five eigenvectors screened by the classifier in differentiating end-stage renal failure

We screened five signature genes, FOS, FOXO3, SIRT1, TP53, and SMARCA4, for clinical diagnostic analysis after Lasso regression analysis and Class cross-validation. ROC curves analyzed the performance of the five pivotal genes in the diagnosis of ESRD (Fig. 7a). The area under the ROC curve (AUC) was calculated to indicate diagnostic efficiency and predictive accuracy (Fig. 7b). The results showed that these five characteristic genes had a high diagnostic value for ESRD samples compared to healthy samples. Specifically, FOS showed the highest diagnostic performance in ESRD samples (AUC: 0.953), followed closely by TP53 (AUC: 0.91), and SIRT1 ranked third (AUC: 0.901). Based on the above data, these five signature genes can be used as potential biomarkers for the development of ESRD.

## Discussion

Cellular senescence has a direct or indirect influence on ESRD, and renal failure also leads to cellular senescence and consequently changes in kidney structure and function. In this study, ESRD-associated and cellular senescence-associated genes were identified from the GEO and CallAge databases, respectively. By differential analysis, 52 specific cellular senescence genes associated with ESRD were obtained.

In the transcriptome analysis network we constructed, ten main hub genes (TP53, MYC, AKT1, SIRT1, FOS, CEBPB, SMARCA4, FOXO3, NFE2L2, and CDKN1B) are involved in the physio-pathological interactions of end-stage renal failure and cellular senescence. SIRT1 is a conserved protein NAD ( +) -dependent deacetylase that is not only an important sensor of energy status. It is also associated with cellular metabolism and cellular senescence regulation [14]. SIRT1 inhibits cellular senescence by delaying age-related telomere attrition and promoting DNA damage repair to maintain genomic integrity [15]. Meanwhile, SIRT1 is a key factor in the development of diabetic nephropathy pathology [16]. In reports of diabetic nephropathy, the acetylation levels of p65 NF-κB and STAT3 are elevated [17], and SIRT1 can confer reno-protective effects by inhibiting renal inflammation, apoptosis, oxidative stress, and fibrosis through deacetylation of STAT3 and p65 NF-κB target proteins to stop the progression of diabetic kidney disease to end-stage renal disease [18, 19]. In addition, the role of SIRT1 in ameliorating oxidative stress is associated with FOXO3 activation [20], calorie restriction maintains renal SIRT1 expression and increases BNIP3 expression by deacetylating FOXO3, promoting mitochondrial autophagy and delaying the effects of aging on the kidney [21]. Vascular aging increases the risk of developing chronic kidney disease, and FOXO3 inactivation is involved in the development of aging-related vascular disease. In an experimental study, FOXO3-regulated ESC-derived vascular cells improved intravascular homeostasis and delayed vascular aging [22]. The deletion of FoxO3 in renal tubular cells during the transition from acute kidney injury to chronic kidney disease decreases autophagy and exacerbates oxidative damage to the kidney, leading to severe fibrosis [23]. CDKN1B overexpression increases genetic damage or induces DNA damage, altering the balance between genomic and tissue aging [24]. In addition to this, other proteins of the CDKN protein family such as Cdkn1a and Cdkn2a are regulated by the p53 and Rb signaling pathways and are elevated in response to DNA damage leading to temporary or permanent cell cycle arrest [25]. Cell cycle or proliferation disorders exacerbate the dysfunction of chronic kidney tissues, and intervention from the aging genome to control it is one of the potential means for ESRD prevention or treatment.

miRNAs are endogenous short-stranded non-coding RNAs that exert their negative regulatory effects at the transcriptional level after binding to complementary sequences of target mRNAs [26]. miRNAs involved in the activation or silencing of target genes play a powerful role in regulating cell growth, differentiation, development, and apoptosis on various cellular activities in terms of epigenetics and intercellular communication [27, 28]. SIRT1 regulates many biological processes associated with aging or apoptosis, including chromatin remodeling, DNA repair, genome stability, autophagy, and telomere maintenance [29]. In our constructed miRNA gene regulatory network, there are 17 examples of SIRT1-associated miRNAs. FOXO1 is a key regulator of vascular growth, and miR-217 induces premature senescence and impairs vascular regeneration by inhibiting SIRT1-FOXO1 function, or increasing SIRT1 expression to reduce cellular senescence and enhance angiogenesis [30]. There may be a functional association between insulin secretion, SIRT1, and miR-9 expression in diabetic patients: miR-9 targets SIRT1 to regulate its expression in insulin-secreting cells. During glucose-dependent insulin secretion, high levels of miR-9 are associated with reduced levels of SIRT1 protein [31]. miR-195 mediates pro-apoptotic effects in cardiomyocytes through the down-regulation of SIRT1 and generation of ROS [32], and there may be a similar mechanism in renal cells. miR-182 is expressed through direct binding to FOXO3 to promote apoptosis in ischemia–reperfusion-induced acute kidney injury [33]; miR-122 enhances renal fibrosis, renal inflammation, and oxidative damage in hypertensive rats by inhibiting the expression of FOXO3 [34]. In addition to this, studies have shown that some drugs can affect renal cell function by influencing the miRNA-FOXO3 regulatory axis. Ellagic acid (EA) exerts reno-protective effects through miR-182/FOXO3 to improve chronic renal failure [35], and curcumin and resveratrol inhibit contrast-induced renal inflammation through the miR-30 / FOXO3 signaling pathway. The pharmacological effects of hub genes and upstream miRNAs have potential therapeutic implications for kidney-related diseases and could open up new avenues for ESRD treatment by manipulating miRNA levels. The same regulatory role as miRNAs can be played by transcription factors (TFs) attached to specific genes, and these proteins play an important role in controlling the transcription of genetic information [36]. In the Metascpae database enrichment analysis, the Hub-targeted collection of 222 TFs was shown to often function as cellular regulatory complexes for the synthesis or modification of genetic material such as chromatin and histones, and these TFs are also involved in the development of chronic kidney disease. For example, SP1 is involved in promoter and cell cycle regulation [37]; overexpression of USF2 leads to urinary protein in non-diabetic mice and induces the development of diabetes [37]; loss of GLIS2 function causes cystic nephropathy characterized by renal atrophy, renal occlusion, inflammation and fibrosis [38], and GLIS2 deficiency is also associated with the development of chronic kidney disease. And GLIS2 deficiency is also associated with an increase in apoptotic renal tubular cells and interstitial infiltration of inflammatory cells [39]. Progression of the disease leads to the development of proteinuria, elevated blood levels of urea nitrogen and creatine, and ultimately to end-stage renal disease [40]. Recent studies have also confirmed the protective effects of GLIS2 on renal cells: GLIS2 inhibits epithelial-mesenchymal transition and apoptosis of renal tubular cells by regulating the β-linked protein signaling pathway in diabetic nephropathy and ameliorates hypertension-induced renal injury by affecting autophagy through upregulation of GLIS2 expression [41, 42].

Of the 359 diseases associated with the 10 Hub genes, nearly 30% (105/359) were associated with tumors or cancers, covering the gastrointestinal, respiratory, urinary, neurological, immune, and circulatory systems. This is consistent with basic cytological physiological cognition, as Cell senescence can cause suppression of tumor cell proliferation and invasion by arresting the cell cycle [43]. In addition to the blood cell carcinomas mentioned above, other diseases of the circulatory system commonly associated with renal failure include cardiovascular abnormalities. Related reports have also confirmed that cardiovascular disease (CVD) is the main cause of morbidity and mortality in patients with end-stage renal disease via hemodialysis [44]. The association of ESRD with CVD is independent of risk factors such as diabetes and hypertension and may be specifically related to uremic toxins, oxidative stress, and endothelial dysfunction [45, 46]. Both ESRD and chronic kidney disease (CKD) treated with conventional hemodialysis or peritoneal dialysis are associated with a high prevalence of left ventricular hypertrophy, and interstitial cell fibrosis [45, 46]. This has been similarly demonstrated in our genetic disease network. The hemodynamic, metabolic, cellular, and molecular mediator abnormalities of myocardial hypertrophy, fibrosis, apoptosis, and capillary degeneration may be associated with myocardial electrical instability, re-entrant arrhythmias, and congestive heart failure [47]. Diabetic nephropathy is the most common cause of chronic kidney disease, and the early identification of patients at risk of developing diabetic nephropathy and the initiation of appropriate treatment is important to improve patient prognosis [48]. Based on the universal nature of cellular senescence in addition to organ function impairment, given that the above diseases see ESRD closely linked to systemic tissues and organs with a wide range of involvement, the management principles of detecting complications and key clinical indicators should be part of the health surveillance of ESRD patients accordingly.

The absolute abundance of eight immune cells and two stromal cells was calculated using MCPcount. Of these, the levels of T cells, Cytotoxic lymphocytes, NK cells, Myeloid dendritic cells, endothelial cells, and Fibroblasts were significantly lower in ESRD, and the levels of CD8 T cells, B lineage, and Monocytic lineage levels were significantly higher. Endothelial cells and fibroblasts are stromal cells that play an important regulatory role in tumor development as an important component of the tumor microenvironment. The results showed that endothelial cells and fibroblasts were significantly lower in the disease group than in the control group, which may be attributed to the elevated levels of CD8 T cells [49] and Monocytic lineage [50] in ESRD patients, especially those associated with cellular immunity. The correlation between Hub genes and immune cells Correlation showed that genes downregulated by Hub in ESRD were positively associated with T cells, NK cells, Cytotoxic lymphocytes Neutrophils, Myeloid dendritic cells, endothelial cells, and Fibroblasts, and with B lineage, CD8 T cells, and Monocytic lineage, a result that also explains the difference in immune cell content between the disease and normal control groups. It has been shown that immunodeficiency and senescence of T cells are associated with the development of ESRD [51–53] and that ESRD patients have a prematurely aged T cell system, which is associated with a T cell-mediated immunodeficiency.

As we presented in our results, oxidative stress, renal inflammation, apoptosis, cellular autophagy, and telomere damage are constantly mentioned and emphasized in functional enrichment and signaling pathway studies. In chronic kidney disease, which is the prophase of end-stage renal failure, Amplification of oxidative stress is present. Excess ROS produced by renal pro-oxidant enzymes and advanced oxidation protein products (AOPPs) produced and accumulated during oxidative stress, oxidized lipids are directly associated with damage to the kidney or kidney units [54]. Meanwhile, the large aggregation of pro-inflammatory cells collected in our immune infiltration results may be caused by the activation and recruitment of nuclear factor κ B transcription factor in a pro-oxidant milieu. In the study of Mikolaj Ogrodnik et al. cellular senescence was the arrest of normal cell division in response to a variety of cellular stresses, DNA damage, pro-inflammatory responses, and telomere shortening [55]. Therefore, we believe that cellular senescence can be seen as the precursor state of renal injury by various cellular stresses represented by oxidative stress, but considering the direct damage to cellular senescence and kidney units by pro-inflammatory factors, cellular senescence can also be considered an accelerator of renal demise.

The limitations of this study are well recognized. Firstly, the sample size was limited by the difficulty of sampling and the limitation of end-stage renal failure samples in the database, we minimized the false positive rate by false discovery rate (FDR) analysis from a statistical point of view. Secondly, due to the heterogeneity of peripheral blood and renal tissue, peripheral blood cannot fully reflect the physiological status of renal cells. However, considering that the main body of our study is the transcriptome, genes are present and expressed in most cells in the human body, and there are also some cell-contained genes in the blood. Therefore, to a certain extent, peripheral blood can be considered a feasible alternative tissue for renal research at the transcriptome level. Finally, this study lacks a multiple-group controlled trial from a gene transcriptome perspective and the accuracy of the results should be re-emphasized in subsequent ex vivo validation trials.

## Conclusions

In summary, we screened ten potential ESRD cellular senescence-associated Hub genes by bioinformatics analysis for function, pathways, relevance, association with miRNA, TFs, drugs, related diseases, and immune regulation, and validated their expression. These study results could expand our understanding of ESRD. In addition, the identification of potential drugs might contribute to the treatment of ESRD.

### Supplementary Information

**Additional file 1: Table S1.** Levels of eight immune cells and two stromal cells in the samples.

## Data Availability

The datasets presented in this study can be found in the GEO database (https://www.ncbi.nlm.nih.gov/geo/query/acc.cgi?acc=gse37171) without any relevant permissions required to access the information from the database.

## Abbreviations

ESRD: End-stage renal disease
GEO: Gene expression omnibus
PPI: Protein–protein interaction
GO: Gene ontology
KEGG: Kyoto encyclopedia of genes and genomes
ROC: Receiver operating characteristic
DCA: Decision curve analysis
AUC: Area under curve
STRING: Search tools for retrieving interacting genes
TFs: Transcription factors
BP: Biological process
CC: Cellular component
MF: Molecular function
CVD: Cardiovascular disease
CKD: Chronic kidney disease
AOPPs: Advanced oxidation protein products
FDR: False discovery rate
